# Broadband Achromatic Hybrid Metalens Module with 100° Field of View for Visible Imaging

**DOI:** 10.3390/s25103202

**Published:** 2025-05-20

**Authors:** Peixuan Wu, Xingyi Li, Yuanyuan Xing, Jiaojiao Wang, Wujie Zheng, Zekun Wang, Yaoguang Ma

**Affiliations:** 1 State Key Laboratory for Extreme Photonics and Instrumentation, College of Optical Science and Engineering, Intelligent Optics and Photonics Research Center, Jiaxing Research Institute, International Research Center for Advanced Photonics, ZJU-Hangzhou Global Scientific and Technological Innovation Center, Zhejiang University, Hangzhou 310027, China; 22330087@zju.edu.cn (P.W.); 0623524@zju.edu.cn (X.L.); 2 Hangzhou Najing Technology Co., Ltd., Hangzhou 310027, China; xyy@najing.cn (Y.X.); wangjj@najing.cn (J.W.); zwj@najing.cn (W.Z.); wangzk@najing.cn (Z.W.)

**Keywords:** hybrid metalens, aberration corrected, wide FOV, visible imaging

## Abstract

Conventional metalenses struggle with chromatic aberration and narrow field of view (FOV), making it challenging to meet the dispersion requirements for large apertures and compensate off-axis aberrations for wide FOV. Here, we demonstrate a hybrid metalens module consisting of five refractive plastic lenses and a polarization-insensitive metalens to achieve broadband achromatic imaging within 400–700 nm and a wide FOV up to 100°. The system exhibits negligible variation in focal length (~1.2%) across the visible range (460–656 nm) and consistently achieves modulation transfer function (MTF) values > 0.2 at 167 lp/mm across all wavelengths and incident angles. We also demonstrate integrated lens modules that capture high-quality images from distances ranging between 0.5 and 4 m without post-processing, showcasing its potential for compact, wide-angle optical systems.

## 1. Introduction

Planar metasurfaces, with their subwavelength-scale meta-atoms enabling unprecedented light manipulation, have catalyzed a paradigm shift in photonic devices [1,2,3]. Among these, phase-gradient metasurfaces are particularly transformative: by encoding spatially varying phase profiles, they enable arbitrary wavefront shaping with unprecedented precision. Over the past decade, a variety of strategies have been explored to realize achromatic metalenses, including dispersion engineering [4,5,6], spatial multiplexing [7,8], computational imaging [9,10], and multi-layer metalenses [11].

Nevertheless, despite their promising applications, metalenses continue to face intrinsic challenges in controlling chromatic aberrations. Although dispersion engineering techniques have successfully expanded achromatic operation into the visible and near-infrared spectral regions [12], fundamental trade-offs remain among numerical aperture (NA), operational bandwidth, and lens aperture size. These trade-offs inherently restrict the practical dispersion management of metalenses featuring high NAs and large apertures with minimized thickness profiles. The central obstacle is the intrinsic limitation in the phase dispersion compensation capacity of individual meta-atoms, which fundamentally constrains the overall polychromatic performance achievable by existing metalens designs [13,14].

To address these intrinsic chromatic aberration constraints, hybrid metalenses—which synergistically combine metasurfaces with conventional refractive lenses—have emerged as a powerful solution [14,15,16]. In such hybrid systems, the metasurface typically serves as a dispersion corrector, compensating for the inherent chromatic dispersion introduced by refractive optics. For instance, Chen et al. demonstrated a compact hybrid metalens display for augmented reality applications, achieving superior resolution and significantly reduced off-axis aberrations across a 30° field of view (FOV), while simultaneously decreasing the overall device track length compared to traditional refractive systems [17].

Although hybrid metalenses efficiently mitigate axial chromatic aberrations [18,19,20], off-axis aberrations such as coma and astigmatism remain problematic, particularly for FOVs exceeding approximately 20° [17,21,22]. Recent advances have explored integrating hybrid metalens designs with computational imaging techniques, exhibiting considerable potential. For example, combining hardware-in-the-loop optimization with convolutional neural-network-based inverse imaging algorithms has yielded performance levels comparable to commercial lenses, extending usable FOV to approximately 60° in the visible spectrum [23]. Shin et al. recently developed a hybrid metalens system composed of two refractive lenses and two metasurfaces, achieving high MTF performance within a 30° FOV in the mid-infrared wavelength [24]. However, incorporating multiple metasurfaces inevitably increased transmission losses.

To further expand the achievable FOV, we propose integrating a single metasurface into a multi-element refractive lens configuration. This hybrid metalens module leverages the metasurface’s precise wavefront-shaping capability, thereby improving aberration correction while maintaining the total of length.

**Figure 1:**
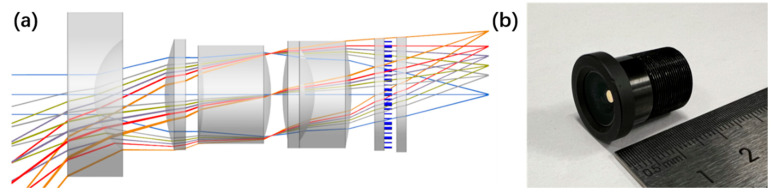
(**a**) Ray-tracing diagram and schematic configuration of the proposed broadband aberration-corrected large-scale hybrid metalens module designed for different FOV angles in the visible spectrum. (**b**) Optical image of the assembled hybrid metalens module.

In this work, we introduce a polarization-insensitive hybrid metalens module designed in the visible spectrum, spanning wavelengths from 460 to 656 nm. As illustrated schematically in Figure 1a, our design integrates five refractive lenses with a single large-area metasurface functioning as an aberration corrector, achieving an F-number of 2 and a full field of view (FOV) of 100°. An optical image of the assembled prototype is shown in Figure 1b.

This hybrid optical architecture strategically leverages refractive components for effective chromatic dispersion control, complemented by the metasurface’s precise wavefront manipulation capabilities, allowing simultaneous correction of axial and off-axis aberrations. Systematic characterization of MTF, wavelength-dependent focal stability, and FOV performance confirms broadband aberration correction. Furthermore, by integrating our hybrid metalens with a commercial CMOS sensor, we demonstrate a compact imaging system capable of capturing high-quality images without the necessity for computational post-processing. Experimental results validate the module’s optical performance, consistently achieving MTF values greater than 0.2 at a spatial frequency of 167 lp/mm. Additionally, the focal length exhibits minimal variation (~1.2%) across the designed spectral range, underscoring the system’s excellent broadband focal stability.

## 2. Materials and Methods

The hybrid metalens module was designed through ray-tracing optimization using commercial optical design software (Zemax OpticStudio 2022, Ansys). The optical configuration consists of five spherical refractive lenses positioned sequentially, followed by a single metasurface element and an infrared filter as the final component. The metasurface’s spatially varying phase profile is mathematically defined by an even-order polynomial series as follows:

$$\varphi \left(r\right)=\sum_{n=1}^{m}a_{n}{\left(r/R\right)}^{2n}$$

where *r* is the radial coordinate of the metasurface, *R* is the normalized radius, $a_{n}$ is the phase coefficients of the even-order polynomial, and *n* is the number of polynomial coefficients in the series. We chose to place the metasurface on the final surface to facilitate assembly while ensuring that the angle of incidence on the metasurface remains small, thus avoiding large phase deviations and low transmittance.

The hybrid metalens module (see Tables S1 and S2 for detailed parameters at the primary wavelength) is optimized to achieve a focal length of 4.13 mm, an F-number of 2, a total optical length of 22.29 mm, and a full field of view (FOV) of 100°, operating effectively over a wavelength range spanning from 460 to 656 nm.

**Figure 2:**
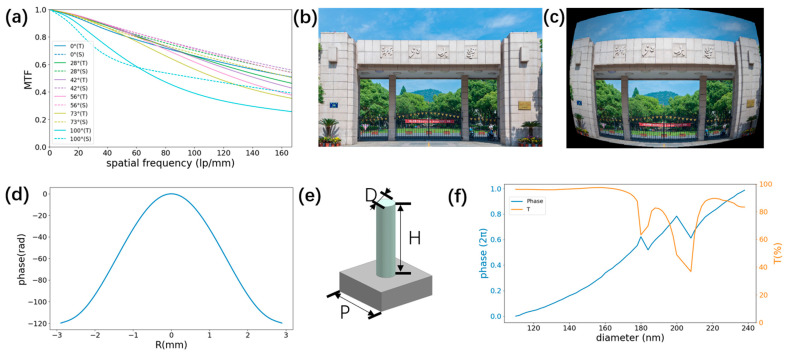
(**a**) Simulated MTF curves of the designed hybrid metalens module for five incident wavelengths and six FOV angles. The solid and dashed lines denote the results of the tangential ray and sagittal ray, respectively. (**b**) Picture of a building as an object to be input into the designed hybrid metalens module. (**c**) Simulated image through the hybrid metalens module. (**d**) Phase radial profile of the metasurface corrector. (**e**) Schematic of a meta-unit of the metasurface corrector. (**f**) Simulated phase shifts and transmission efficiencies of a series of circular SiNx pillars.

Figure 2a illustrates the simulated MTF of the optimized hybrid metalens module, evaluated across five distinct wavelengths and six FOV angles, thereby quantifying spatial-frequency-dependent image contrast. The simulated curves demonstrate consistent MTF values surpassing 0.2 at the critical spatial frequency threshold of 167 lp/mm, meeting the fundamental requirement for discernible image contrast as established in optical system evaluations. For direct comparison, Figure S1 presents the MTF response of the original lens module, scaled to the same overall length as the hybrid design. Compared with the initial module total length of 21 mm, our proposed design achieves an improvement in MTF while maintaining a nearly identical overall length of 22.29 mm. Furthermore, Figure S2 shows that after removing the metasurface and refocusing, the simulated MTF of the system drops significantly. It is evident that the metasurface plays an important role in aberration correction in the module we designed while maintaining the total of length.

A standardized 780 × 480 pixel test pattern (Figure 2b) was employed to validate imaging fidelity, with the reconstructed image (Figure 2c) retaining essential scene details despite observable edge blurring and distortion—characteristics attributable to residual wavefront errors in broadband operation.

Phase correction is achieved via a radially optimized metasurface (aperture = 5.79 mm; Figure 2d) fabricated from silicon nitride (SiNx) nanopillars on a glass substrate. SiNx was selected for its subwavelength thickness, which minimizes absorption losses, and for its compatibility with electron-beam lithography, enabling high-precision nanopatterning. As shown in Figure 2e,f, each unit cell comprises a cylindrical SiNx pillar with diameter D varying from 110 nm to 240 nm, height H = 700 nm, and lattice pitch P = 340 nm. This geometry provides a continuous phase coverage of 2π while maximizing transmission at the design wavelength (λ = 546 nm). The phase profile is discretized at the 340 nm lattice constant to ensure accurate sampling and seamless phase continuity across the aperture. Broadband phase-response characteristics are detailed in Figure S3.

**Figure 3:**
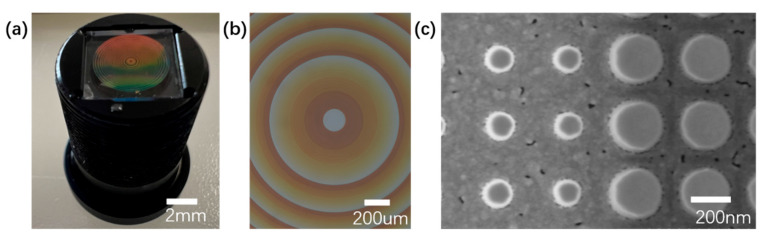
(**a**) Optical image of the assembled hybrid metalens module. (**b**) Optical microscope image of the metasurface corrector. (**c**) Scanning electron micrographs of metasurface corrector.

In our work, electron-beam lithography (EBL) was used for the fabrication of the metasurface. If there is a need for mass production in the future, nanoimprint lithography [25,26] or photolithography [27] will be adopted. The metalens was fabricated using a 700 nm thick SiNx film, formed via plasma-enhanced chemical vapor deposition (PECVD) equipment (Leuven P200A). The refractive index, measured using an ellipsometer (UVISEL), was determined to be 2.39 at a central wavelength of 546 nm. Subsequently, the film underwent spin coating with a ma-N2405 electron beam resist from Micro Resist Technology. The metalens pattern was then generated using EBL (Raith Voyager) and developed in ma-D 525. Following this, the designed pattern array acted as an etch mask for transferring its design onto the wafer by using inductively coupled plasma-reactive ion etching equipment (Samco RIE-230IP). Finally, any remaining traces of the etch mask were eliminated through plasma etching. For the fabrication of the refractive lens, single-point diamond turning was utilized. We assembled the refractive lens and the flat metasurface into a customized lens mount, creating a hybrid metalens module. Figure 3a presents an optical image of the fabricated hybrid metalens module with a metasurface on the top. Figure 3b shows the optical image under a five-fold microscope revealing structural uniformity across the device, and Figure 3c displays a scanning electron micrograph featuring two segments of SiNx pillars from the metasurface. The small dark spots in Figure 3c are attributed to material residues that are formed when coating the uneven substrate for SEM imaging, which do not significantly impact the module’s performance.

## 3. Results

**Figure 4:**
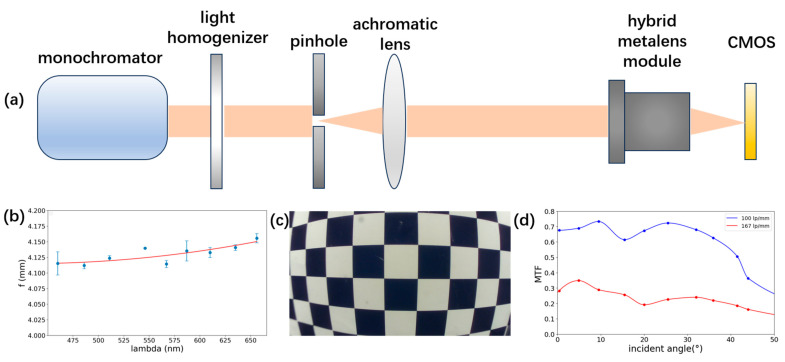
(**a**) Scheme of the optical setup for measuring the focal length changing at different wavelengths. (**b**) Focal length at different wavelengths. (**c**) Image of a checkerboard. (**d**) Measured polychromatic MTF curve as a function of FOV angles at the spatial frequency of 100 and 167 lp/mm.

The optical performance of the fabricated hybrid metalens module was evaluated by coupling it to a commercial CMOS sensor (3 μm pixel pitch). We first quantified focal length stability across the designed spectrum. Figure 4a depicts the experimental setup used for these measurements: a monochromator, followed by a light homogenizer to ensure uniform beam profile. The beam then passed through a precision pinhole and an achromatic collimator before entering the hybrid metalens, which was mounted on a high-resolution translation stage. The image plane is defined by the CMOS detector. Figure 4b summarizes the measured focal lengths as a function of wavelength, overlaid with a polynomial fit. The data demonstrate remarkable chromatic focal stability, with the focal length varying by only ~50 μm across the 460–656 nm range—equating to a deviation of just 1.2% from the nominal design value of 4.13 mm.

Next, we evaluated the effective imaging field of view by imaging a standardized checkerboard target (square pitch = 21 mm; see Supplementary Figure S4). Figure 4c shows the captured image of an 8.5 × 6 grid placed at an object distance of 90 mm. The captured image exhibits uniformly sharp edges and minimal distortion or blur across the entire image plane, verifying the optical quality and uniformity of the imaging performance. From the maximum resolved diagonal span of over 218 mm at this distance, we infer a full-angle FOV of approximately 100°.

Finally, we conducted a polychromatic MTF evaluation using the Slanted Edge Method (see Supplementary Information for experimental details). Figure 4d compares the measured MTF at spatial frequencies of 100 lp/mm and 167 lp/mm for both on-axis and selected off-axis field positions. Averaged over the entire imaging field, the MTF reached 0.63 at 100 lp/mm and 0.24 at 167 lp/mm, and at the maximum-field position, the MTF still maintained values of 0.364 and 0.161 at 100 lp/mm and 167 lp/mm, respectively. Minor deviations from the theoretical simulations are attributed to fabrication tolerances and alignment uncertainties; nonetheless, the experimental MTF performance confirms the module’s strong aberration correction and practical imaging quality.

To experimentally demonstrate and verify the practical imaging capabilities of the developed hybrid metalens module, we implemented a compact visible-light imaging system by directly integrating the hybrid metalens module with the CMOS sensor (prototype details are provided in Supplementary Figure S5). The resulting hybrid metalens module system was comprehensively evaluated under laboratory conditions using a carefully selected combination of imaging targets. Specifically, imaging assessments utilized the ISO12233 resolution chart alongside a 24-patch ColorChecker array, further complemented by chromatically distinct objects (red scissors, blue card, and green tape) to rigorously test and clearly visualize color reproduction and contrast performance.

**Figure 5:**
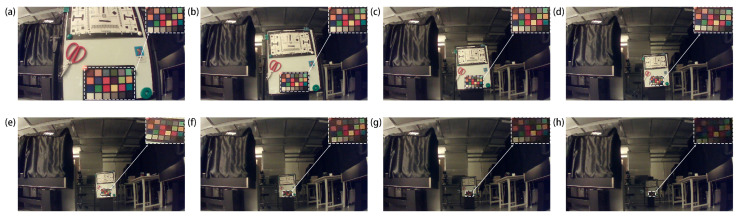
(**a**–**h**) Images captured by the assembled hybrid metalens module in the lab, with object distances of, respectively, 0.5, 1, 1.5, 2, 2.5, 3, 3.5, and 4 m.

Figure 5a–h present unprocessed images captured at object distances ranging from 0.5 m to 4 m, recorded without any computational correction or post-processing. In each frame, the 24-patch ColorChecker chart has been uniformly scaled to 300 × 200 pixels and positioned in the upper-right corner. For object distances up to 2 m, the chart remains sharply resolved; beyond this range, its apparent blur increases commensurate with the reduced pixel coverage. Across all photos, the images consistently exhibit crisp edge definition, faithful color reproduction, and high-contrast resolution. These results demonstrate the hybrid metalens module’s exceptional optical stability and its ability to maintain image clarity and color accuracy over a wide range of working distances—affirming its suitability as a compact, all-optical solution for diverse practical imaging applications.

## 4. Conclusions and Discussion

In summary, this work resolves two fundamental limitations of conventional metalenses—chromatic dispersion and constrained FOV—through a hybrid metalens module combining five achromatic refractive elements with a polarization-insensitive metasurface phase corrector. The synergistic design employs refractive components for broadband dispersion compensation (460–656 nm) while utilizing the metasurface for high-precision wavefront correction, achieving both minimal wavelength-dependent focal shift (1.2% of 4.13 mm design length) and wide 100° FOV. Ray-trace-optimized metasurface phase profiles, parameterized through polynomial functions, enable MTF performance exceeding 0.2 at 167 lp/mm across majority field angles. Experimental characterization validates these metrics through quantitative MTF analysis and qualitative imaging assessments, with checkerboard pattern resolution and multi-distance scene capture confirming practical applicability in real-world imaging scenarios. While minor MTF discrepancies between simulated and experimental results are attributed to current sub-micron fabrication constraints and alignment tolerances, the demonstrated performance shows significant progress in metalens-based imaging systems.

From an industrial standpoint, the hybrid metalens module represents a strategically engineered convergence of conventional refractive optics and emerging metasurface technologies. By leveraging the maturity and reliability of traditional lens manufacturing alongside the rapid scalability of planar nanofabrication, this design paradigm offers a viable pathway toward high-volume, cost-efficient production of advanced optical systems. The inherently compact architecture of the hybrid module, coupled with its seamless compatibility with standard CMOS imaging platforms, substantially reduces the complexity and cost of integration across a broad range of industrial applications. As precision in nanofabrication continues to improve, particularly in the patterning of large-area metasurfaces, this hybrid optical framework is poised to enable a new class of ultra-miniaturized, high-performance imaging devices. Such advancements will accelerate the transition of metasurface-based innovations from laboratory research to practical deployment in real-world scenarios.

Looking ahead, key developmental directions include enhancing the fabrication of large-aperture metasurfaces, refining broadband achromatic design methodologies, and incorporating computational imaging techniques. These efforts aim to significantly elevate imaging performance under constraints such as limited fabrication condition, wide spectral bandwidth, or environmental noise. The hybrid metalens’ adaptability to diverse wavelengths and scalability for larger apertures positions it as a versatile platform for next-generation optical systems, spanning consumer electronics, surveillance, automotive sensors, and beyond.

## Data Availability

All data that support the findings of this study are included within the article and Supplementary Materials.

## Supplementary Materials

The following supporting information can be downloaded at: https://www.mdpi.com/article/10.3390/s25103202/s1, Note S1: Slanted Edge Method for MTF test [28]; Table S1: The parameters of the refractive lenses; Table S2: The parameters of the metasurface; Figure S1: The simulated MTF response characteristics of the system before introducing the metasurface; Figure S2: The simulated MTF response characteristics of the system after removing the metasurface and refocusing: Figure S3: The broadband phase-response characteristics of the metasurface; Figure S4: The optical images of the checkerboard (a), ISO12233 resolution chart (b), and 24-patch ColorChecker (c); Figure S5: The optical images of the visible-light imaging system by integrating the hybrid metalens module with a commercial CMOS detector.

## Abbreviations

The following abbreviations are used in this manuscript:

FOV	field of view
MTF	modulation transfer function
SiNx	silicon nitride
