# Supplementary material for: Broadband Achromatic Hybrid Metalens Module with 100° Field of View for Visible Imaging
Source: Sensors (Basel). 2025 May 20;25(10):3202. doi: 10.3390/s25103202 (PMC12115752; doi:10.3390/s25103202)
Supplement: Supplementary file 1 [file sensors-25-03202-s001.zip › sensors-3582553-supplementary.pdf]

Supporting Information for Broadband Achromatic Hybrid Metalens Module with 100° Field-of-View for Visible Imaging

Note S1. Slanted Edge Method for MTF test

The Slanted Edge Method is a standard technique for quantifying the MTF of optical systems [1]. It consists in imaging an edge (we use the checkerboard as the object) onto the detector, slightly tilted with regard to the rows (or the columns). Then analyze the edge's spread function (ESF) across the image, compute its derivative to get the line spread function (LSF), and apply a Fourier transform to derive the MTF curve, showing contrast retention at varying spatial frequencies.

Tabel S1. The parameters of the refractive lenes

number	Radius	thickness	nd, nv
1	-92.795<br>3.501	1.279	1.69,49.2
2	8.614<br>infinite	1.083	2.00,29.1
3	-10.922<br>-7.310	3.251	1.85,32.3
4	10.212<br>-4.098	1.698	1.67,47.2
5	-4.098<br>-12.144	1.668	1.95,18.0

Tabel S2. The parameters of the metasurface

R	a1	a2	a3	a4	a5
1mm	-3.668E1	4.511E0	-4.426E-1	4.412E-2	-2.088E-3

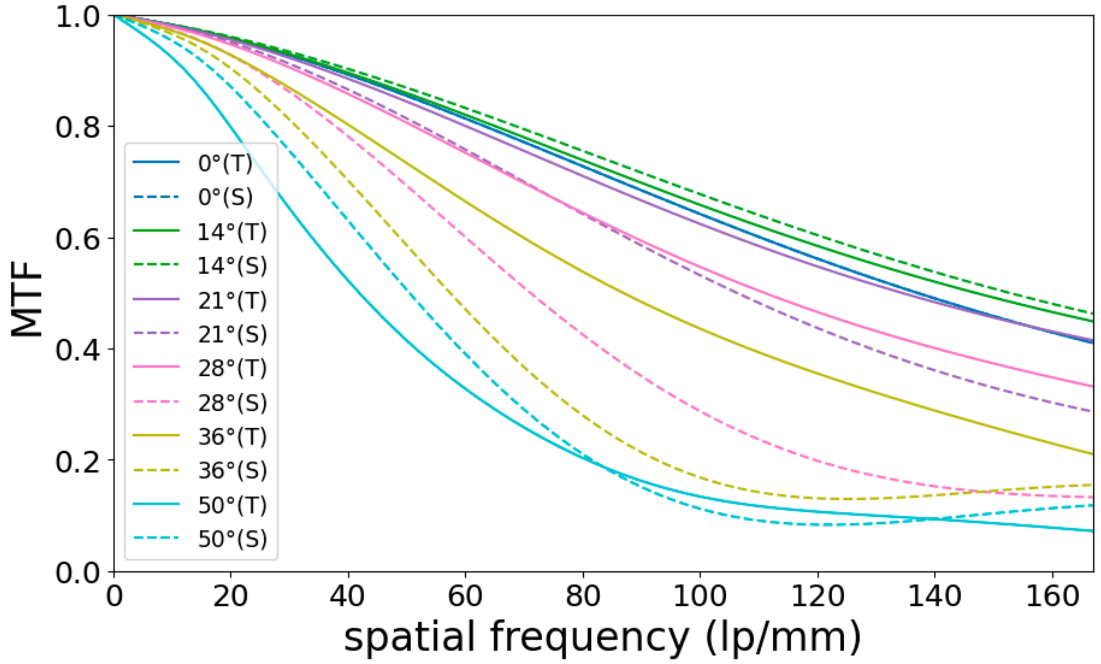

Figure S1. The simulated MTF response characteristics of system before introducing metasurface.

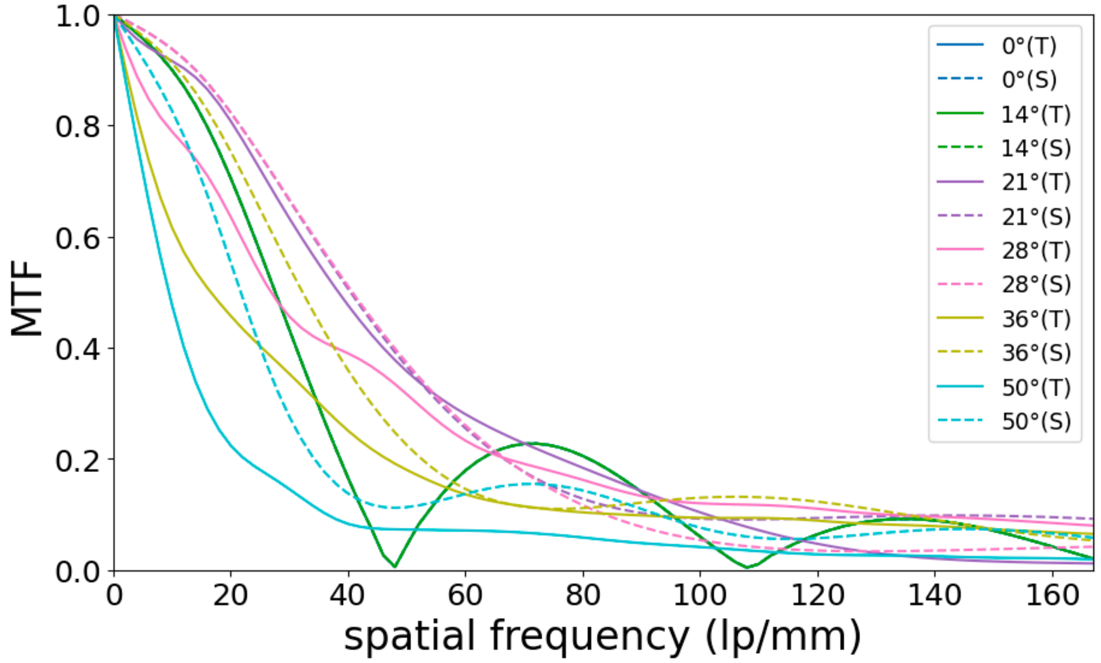

Figure S2. The simulated MTF response characteristics of system after removing the metasurface and refocusing.

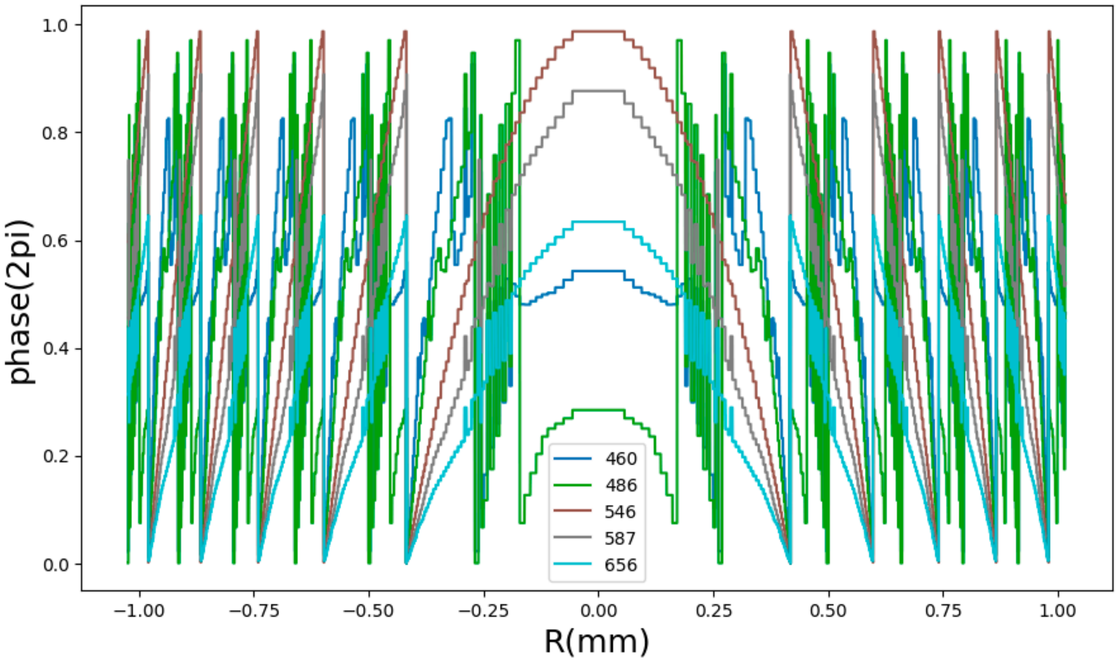

Figure S3. The broadband phase response characteristics of metasurface.

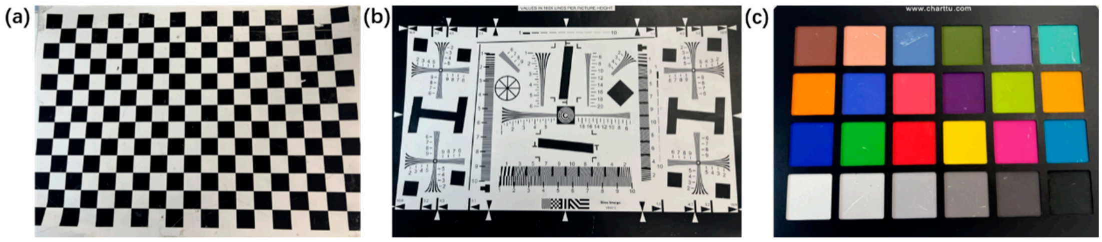

Figure S4. The optical images of the checkerboard (a), ISO12233 resolution chart (b) and 24-patch ColorChecker (c).

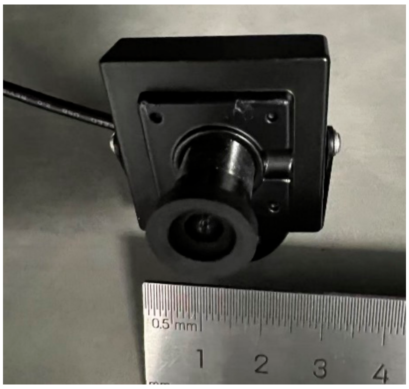

Figure S5. The optical images of the visible-light imaging system by integrating the hybrid metalens module with a commercial CMOS detector.

Reference

[1] Estribeau M, Magnan P. Fast MTF measurement of CMOS imagers using ISO 12333 slanted-edge methodology[C]//Detectors and Associated Signal Processing. SPIE, 2004, 5251: 243-252.
